# Research to support evidence-informed decisions on optimizing the contributions of nursing and midwifery workforces

**DOI:** 10.1186/s12960-020-0459-0

**Published:** 2020-03-20

**Authors:** James Buchan, James Campbell, Carey McCarthy

**Affiliations:** 1 Human Resources for Health, BMC, London, United Kingdom; 2 University of Technology, Sydney, Australia; 3 Health Workforce Department, World Health Organization, Geneva, Switzerland

*Human Resources for Health*, in its longstanding collaboration with the World Health Organization, is strongly committed to making a sustained contribution to improving the evidence base on the value and contribution of the nursing and midwifery workforce. This is reflected in the Board’s composition and in our 2019 publications. Four of the Associate Editorial team, and eight additional board members are drawn from the two professions. In 2019, the journal published more than 60 papers covering nursing and/or midwifery workforce issues: 20 with a specific and detailed focus and more than 40 others where the two professions were a significant component of the overall analysis.

We anticipate no diminution of our coverage in 2020 and beyond. Nurses and midwives represent the largest component of the health professional workforce in nearly all locations and countries. They are critical to the achievement of Universal Health Coverage, and to improving health globally; investment in this workforce can also contribute to improved gender equality, and to building stronger economies [1].

The year 2020 has a specific resonance within the professions, having been designated by WHO as the Year of the Nurse and the Midwife. Two key reports are in development, covering “The State of the Worlds Nursing 2020” and “State of the Worlds Midwifery 2021”, We aim to make our own contribution across this special year through a new Call for papers [2], raising the profile of the professions, and improving the evidence base of their impact and potential.

Much work remains to be done to improve and expand this evidence base on the nursing and midwifery workforces. We went “live” with the Call in November 2019, and we have already published the first papers, with more in the pipeline. The papers that we have published include:

Claudia Maier’s paper examines the growth in nurse prescribing across Europe [3]. Regulation and legislation enabling nurses and midwives to prescribe is of the main enablers to support these staff into advanced and autonomous practice. The cross-country comparative analysis, drawing from an expert survey and an OECD review, demonstrated that, as of 2019, a total of 13 countries in Europe have adopted laws on nurse prescribing, of which 12 apply nationwide (Cyprus, Denmark, Estonia, Finland, France, Ireland, Netherlands, Norway, Poland, Spain, Sweden, United Kingdom (UK)) and one regionally, to the Canton Vaud (Switzerland). Eight of these countries adopted laws since 2010. The extent of prescribing rights ranged from nearly all medicines within nurses’ specialisations (Ireland for nurse prescribers, Netherlands for nurse specialists, UK for independent nurse prescribers) to a limited set of medicines (Cyprus, Denmark, Estonia, Finland, France, Norway, Poland, Spain, Sweden).

“Decent work” is at the core of any employment contract which aims to support staff to perform at the optimum level, feel valued, and be retained and motivated. Aristizabal and colleagues [4], used repeated cross-sectional study of data from the population-based National Occupation and Employment Survey to examine the trends in the level of precarious employment for nurses in Mexico. Precarization was analysed using a quantitative approach to assess its prevalence and geographic distribution between 2005 and 2018. Indicators used included (a) the percentage of people with no written contract, (b) the percentage of people with incomes lower than minimum wage, (c) the percentage of nurses without social security, and (d) the percentage of nurses without social benefits. The study concluded that precarization had increased over the period and now covered more than half (53%) of nurses by 2018.

Other papers in the pipeline, which were submitted for the Call, include new evidence on staff burnout, labour markets, unemployment, and on staffing data. Please check periodically on the journal site to catch up on the latest publications.

We intend holding the Call open across the year. The Year of the Nurse and the Midwife will raise awareness, gather data, promote dialogue and facilitate policy decisions and national investment, but we also need to work together beyond the year end. The Journal will contribute in 2020 and will then continue to generate more research and analysis that shines a light on the contribution of the professions, improves our understanding of their labour markets, and helps delineate the scope to increase their effectiveness, expand their role and impact, and raise their profile with policy makers. In that regard, every year is the year of the nurse and the midwife.

## Data Availability

Not applicable.
